# A Molecularly Imprinted Fluorescence Sensor for the Simultaneous and Rapid Detection of Histamine and Tyramine in Cheese

**DOI:** 10.3390/foods14091475

**Published:** 2025-04-23

**Authors:** Xinpei Li, Zhiwei Wu, Hui Cao, Tai Ye, Liling Hao, Jinsong Yu, Min Yuan, Fei Xu

**Affiliations:** Shanghai Engineering Research Center of Food Rapid Detection, School of Health Science and Engineering, University of Shanghai for Science and Technology, Shanghai 200093, China; Lee_xp97@163.com (X.L.); william-wzw@outlook.com (Z.W.); caohuian@126.com (H.C.); taiye@usst.edu.cn (T.Y.); holiday_hao1988@126.com (L.H.); yujinsong84@163.com (J.Y.); xufei8135@126.com (F.X.)

**Keywords:** histamine, tyramine, molecular imprinting, fluorescence sensor, dairy products, rapid detection

## Abstract

Based on dual-template molecular imprinting polymerization technology, a fluorescent molecularly imprinted polymer doped with CdSe/ZnS quantum dots was developed to construct a “Turn-on” fluorescence sensor for the rapid, sensitive, and specific detection of two biogenic amines. The biogenic amines bind to the quantum dots, which eliminates surface defects and enhances the fluorescence emission intensity of the quantum dots. By optimizing both the polymerization and detection processes, the results demonstrate that the sensor can detect biogenic amines within the range of 0.01–10 mmol/L, with a low detection limit of 14.57 μmol/L and a detection time of only ten minutes. Moreover, the sensor is cost-effective and does not require specialized instrument operation, offering a practical approach for the rapid detection of biogenic amines in complex food matrices. This study advances the development of simultaneous recognition and rapid detection technologies for multiple target molecules.

## 1. Introduction

Biogenic amines (BAs) are a class of small-molecule, biologically active nitrogen-containing organic compounds [1]. In pure dairy products, the levels of biogenic amines are typically low. However, during the production of fermented dairy products such as yogurt and cheese, processes like fermentation can significantly increase the concentration of biogenic amines [2], making their content an important criterion for evaluating the quality of fermented foods. Among these, tyramine and histamine are the most abundant and toxic biogenic amines [3]. As common low-molecular-weight monoamines, histamine (His) and tyramine (Tyr) play specific physiological roles; for example, moderate levels of histamine in the body enhance vascular permeability [4] and lower blood pressure. However, the excessive accumulation or ingestion of biogenic amines can lead to severe poisoning symptoms [5]. To address this issue, various countries have established regulatory limits for biogenic amine levels in different foods. The U.S. Food and Drug Administration (FDA) specifies that histamine levels in food must not exceed 50 mg/kg, while tyramine levels must remain below 100 mg/kg [6]. The European Union sets a limit range of 50–100 mg/kg for biogenic amines in fermented foods [7]. Given that histamine and tyramine are among the most toxic biogenic amines [8], reducing their levels in dairy products is essential for ensuring food safety and minimizing the risk of biogenic amine poisoning.

High-performance liquid chromatography (HPLC) [9] is a widely used analytical technique for detecting biogenic amines, characterized by high sensitivity and high-throughput capabilities [10]. However, its application in analyzing complex food matrices is limited due to the need for intricate sample pretreatment procedures [11] and specialized operational skills. In complex food samples [12], interfering substances may significantly compromise the recovery and purity of target analytes. Cheese, with its high fat and protein content [13], presents more demanding sample processing requirements compared to other fermented foods. To address this challenge, molecularly imprinted polymers (MIPs) [14] have been extensively utilized. MIPs are synthetic analogs of the natural antibody–antigen system [15], featuring stable and abundant imprinted cavities [16] formed through crosslinkers and functional monomers. These cavities adhere to the “lock-and-key” principle [17], exhibiting complementarity to their analytes in terms of shape, size, specific binding sites, and functionality, thereby conferring exceptional selectivity. Owing to their simple preparation process, low cost, excellent stability, and high specificity [18], MIPs have garnered significant attention and found broad applications in areas such as sample separation [19], biosensors [20], and sample pretreatment [21].

Recent studies have explored the use of MIPs for the adsorption and detection of biogenic amines. For instance, Wang et al. [22] developed a method to produce a molecularly imprinted fluorescence sensor with high sensitivity and selectivity for tyramine; the limit of detection was 21 μg/L and the sensor selectively detected tyramine in spiked rice wine samples. Feng et al. [23] developed a facile and cost-effective MIP-based fluorometric assay to directly quantify histamine with a detection limit of 1.80 μM, and this method was successfully used to detect histamine in spiked diary milk with a recovery rate of more than 85%. Yao et al. [24] developed a method combining molecular imprinting technology with metal–organic frameworks to quantify tyramine in food samples. Zhang et al. [25] designed a fluorescence probe based on red-emitting biomass-derived carbon dots (r-BCDs@MIPs) for the sensitive detection of tyramine in fermented meat products. Wang et al. [26] explored a ratiometric imprinted fluorescence sensor based on blue/orange MXene quantum dots with a limit of detection of 21.9 nM for fluorescence detection and 92.2 nM for visual detection; the method was validated for the detection of mackerel, Atlantic cod, and canned tuna with excellent recoveries. However, these methods necessitate detection times exceeding 20 min, even reaching 240 min, making them unable to fulfill the requirement for rapid detection. Furthermore, cheese samples are intricate and fraught with numerous interfering factors, thereby necessitating the development of a more rapid and precise method for detecting biogenic amines in complex samples.

In this study, we developed a fluorescence sensor based on molecularly imprinted polymers (MIPs), leveraging the fluorescence enhancement effect of CdSe/ZnS quantum dots to enable the rapid and simultaneous detection of histamine and tyramine in cheese. The results showed that the synthesized MIP-based fluorescence sensor exhibited outstanding sensitivity and selectivity for the simultaneous detection of these two biogenic amines, with a limit of detection (LOD) of 14.57 μmol/L. Furthermore, when applied to cheese samples, the sensor achieved excellent recovery rates (99.46–103.02%) and high precision (relative standard deviation (RSD) < 6.16%), validating its capability for the sensitive and reliable detection of histamine and tyramine in complex food matrices. This development holds significant promise for the rapid on-site monitoring of biogenic amines in food safety applications.

## 2. Materials and Methods

### 2.1. Chemicals and Materials

Histamine (≥97%), tyramine (≥98%), ethylene glycol dimethacrylate (EGDMA) (≥98%), tetraethyl orthosilicate (≥99%), mercaptoacetic acid (≥99%), and methacrylic acid (≥99%) were all purchased from Sigma-Aldrich (St. Louis, MO, USA). Tryptamine (≥98%), phenylethylamine hydrochloride (≥99%), Dopamine (≥98%), putrescine (≥99%), histidine (≥98%), and tyrosine (≥98%) were all purchased from Shanghai Macklin Biochemical Technology Co., Ltd (Shanghai, China). Azobisisobutyronitrile was purchased from Tianjin Guangfu Fine Chemical Research Institute (Tianjin, China). Anhydrous ethanol (≥99.7%), ammonia water (25.0~28.0%), methanol (≥99.7%), acetic acid (≥99.8%), cadmium chloride (≥99%), selenium dioxide (≥99%), sodium borohydride (≥98%), and sodium hydroxide (≥96%) were all purchased from China National Pharmaceutical Group Chemical Reagent Co., Ltd. (Shanghai, China). All the water used was ultra-pure water prepared in the laboratory using the Mill-Q system. The cheese powder used in the experiment was Crystal Farm Parmesan cheese, purchased from the local supermarket, stored in a refrigerator at 4 °C, and used within the quality guarantee period. The reagents used in this study were of analytical grade and chromatographic grade.

The fluorescence spectra were measured using a Shimadzu RF-6000 FL fluorescence spectrometer (Shimadzu, Kyoto, Japan). The high-performance liquid chromatograph (Waters e2695) was purchased from Waters Corporation, Milford, CT, USA. The chromatographic conditions were as follows: Mobile phase A was composed of 90% acetonitrile and 10% aqueous phase (prepared by dissolving 0.77 g ammonium acetate in 10 mL of acetic acid solution and diluting to 1 L). Mobile phase B consisted of 90% aqueous phase and 10% acetonitrile. The gradient elution program was conducted according to the national standard GB5009.208-2016 [27], and the detailed information can be found in the Supplementary Materials.

### 2.2. Preparation of CdSe/ZnS Quantum Dots

Accurately weigh 456.7 mg of CdCl_2·5_H_2_O and dissolve it in 100 mL of ultrapure water. Subsequently, add 300 μL of mercaptoacetic acid dropwise to the solution and adjust the pH to 8 using a 1 mol/L NaOH solution. Perform magnetic stirring at room temperature for 30 min, followed by sequentially adding 100 mg of NaBH_4_ and 111 mg of SeO_2_. Conduct nitrogen purging for 20 min, then heat the reaction mixture in an oil bath at 100 °C for 2 h. During this period, observe the gradual color change in the reaction solution from light yellow to orange-red. Upon completion of the reaction, rapidly cool the solution in ice water to obtain the CdSe quantum dot solution.

Dissolve 136.3 mg of ZnCl_2_ in 100 mL of deionized water. Add 300 μL of mercaptoacetic acid dropwise and adjust the pH of the mixture to 8 with a 1 mol/L NaOH solution. Stir magnetically at 600 r/min for 30 min, then add 240 mg of Na_2_S·9H_2_O to prepare the ZnS precursor solution. Immediately transfer this solution into the previously synthesized CdSe quantum dot solution and react in an oil bath at 100 °C for 2 h. After the reaction is completed, rapidly cool the mixture in ice water. To precipitate the quantum dots, add three times the volume of anhydrous ethanol, collect the precipitate by centrifugation, and dry under vacuum to obtain the final CdSe/ZnS quantum dots.

### 2.3. Preparation of Imprinted Polymer Fluorescent Sensor with Histamine and Tyramine as Dual Templates

Accurately weigh 11.1 mg of histamine and 13.7 mg of tyramine, and dissolve them in anhydrous ethanol to prepare 10 mmol/L stock solutions. Subsequently, transfer 5 mL of each stock solution into a 250 mL round-bottom flask. Add 50.9 μL of methacrylic acid (0.6 mmol) as the functional monomer and stir the mixture for 1 h at room temperature to form the pre-polymerization complex of template molecules and functional monomers. Dissolve 10 mg of CdSe/ZnS in 5 mL of deionized water and add this solution to the round-bottom flask. Stir the resulting mixture in the dark for 1 h to ensure complete interaction between the template molecules, quantum dots, and functional monomers.

After pre-polymerization, sequentially add 200 mg of SiO_2_ as the imprinting carrier, 2.01 mL of EGDMA (10 mmol) as the crosslinker, 5 mL of ethanol as the solvent, and 10 mg of AIBN as the initiator to initiate the imprinting polymerization process. To eliminate oxygen interference during polymerization, perform nitrogen purging for 20 min and then seal the flask. Place the flask in a 60 °C oil bath and stir continuously in the dark for 24 h. After the polymerization is completed, centrifuge the reaction mixture at 10,000 rpm for 10 min to collect the precipitate. Elute the template molecules (histamine and tyramine) using a methanol solution containing 10% acetic acid. Dry the product under vacuum at 40 °C for 12 h to obtain CdSe/ZnS-MIP.

The preparation procedure for the non-imprinted polymer (CdSe/ZnS-NIP) is identical to that of the imprinted polymer, except that histamine and tyramine are excluded as template molecules during the synthesis process.

### 2.4. Detection of the Sensitivity of CdSe/ZnS-MIP to Biogenic Amines

A mixed solution of histamine and tyramine with a concentration range of 0.2–10 mmol/L was prepared. One milliliter of each concentration solution was accurately measured, and 1 mL of CdSe/ZnS-MIP was added to each sample. The resulting mixtures were shaken for 10 min at a constant temperature of 25 °C to ensure complete interaction between the biogenic amines and the polymer. Subsequently, the fluorescence spectra of the samples in the range of 600–650 nm were recorded using a fluorescence spectrophotometer equipped with an R-60 filter and an excitation wavelength of 340 nm.

For quantitative analysis, the blank fluorescence intensity (F_0_) of the fluorescent molecularly imprinted polymer was used as the reference standard. The fluorescence enhancement rate (ΔF/F_0_) of the sensor was calculated based on the difference between the fluorescence response peak (F_1_) of different concentrations of histamine, tyramine, and mixed solutions to CdSe/ZnS-MIP and the blank fluorescence value (ΔF = F_1_ − F_0_). The fluorescence growth rates of the fluorescent molecularly imprinted polymer in response to different concentrations of histamine, tyramine, and mixed solutions were plotted on the y-axis, while the concentrations of histamine, tyramine, and mixed solutions were plotted on the x-axis. Linear regression analysis was performed to determine the linear detection range.

### 2.5. Specific Detection of Structural Analogs by CdSe/ZnS-MIP

To verify the selective recognition capability of CdSe/ZnS-MIP for specific biogenic amines, fluorescence responses were assessed for structural analogs of histamine and tyramine, including tryptamine, dopamine, phenylethylamine, putrescine, histidine, and tyrosine, all prepared at a concentration of 1 mmol/L. These biogenic amines and amino acids possess structural similarities to the target molecules and may thus influence the selective recognition performance of CdSe/ZnS-MIP. One milliliter of MIP and NIP was individually combined with 1 mL of each biogenic amine solution, followed by shaking at 25 °C for 10 min. Subsequently, fluorescence spectra were acquired using a fluorescence spectrophotometer. To ensure the reliability and accuracy of the experimental results, three independent replicate experiments were conducted for each biogenic amine.

### 2.6. Application of CdSe/ZnS-MIP in Cheese Samples

Cheese was utilized as a real-world sample to assess the sensor’s capability in detecting two biogenic amines (histamine and tyramine) in complex matrices. Three groups of 10 g cheese samples were individually combined with 5 mL of histamine and tyramine solutions at varying concentrations, followed by incubation for 30 min at room temperature. Subsequently, 20 mL of 5% trichloroacetic acid was added to each cheese sample and shaken for 30 min to extract biogenic amines. The resulting mixtures were centrifuged at 5000 rpm for 10 min, and the supernatants were collected. The precipitates were re-extracted under identical conditions. The supernatants obtained from the two extractions were pooled and diluted with trichloroacetic acid to a final volume of 50 mL, producing cheese extracts containing different concentrations of biogenic amines, respectively. A blank sample devoid of biogenic amines was also prepared concurrently. The prepared samples reacted with CdSe/ZnS-MIP and NIP, and their fluorescence responses were recorded. Based on these responses, the concentrations of biogenic amines in the extracts were quantified. To confirm the accuracy of the sensor, the biogenic amine concentrations in the extracts were further determined using HPLC.

## 3. Results and Discussion

### 3.1. Preparation Principle and Characterizations of CdSe/ZnS-MIP

**Figure 1:**
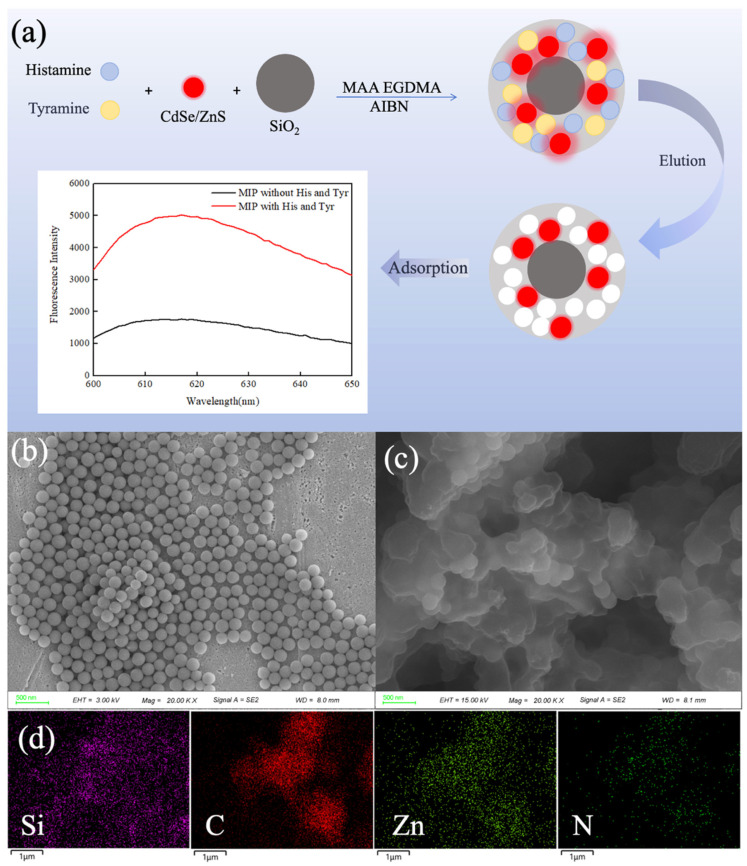
(**a**) Principle diagram and characterization of CdSe/ZnS-MIP; SEM images of (**b**) SiO_2_ and (**c**) CdSe/ZnS-MIP; EDS images of (**d**) CdSe/ZnS-MIP. (His and Tyr: abbreviations for histamine and tyramine).

The sensor integrates molecularly imprinted technology with fluorescent quantum dot technology. Silica microspheres are employed as carriers, and double-template imprinted polymers are formed on their surfaces via surface polymerization, as depicted in Figure 1a. Compounds containing amino groups can enhance the fluorescence intensity of quantum dots [28] by binding to their surface. This interaction eliminates surface defects, thereby increasing the fluorescence emission intensity of the quantum dots [29]. The degree of fluorescence enhancement is utilized to sensitively detect histamine and tyramine.

In the pre-polymerization stage, the functional monomers were bonded to the template molecules via hydrogen bonding [30]. The identification of the functional monomers and template molecules was achieved using UV absorption spectroscopy. The theoretical value presented in Figure S1 corresponds to the superimposed absorption spectra of the monomer and the template molecule, whereas the measured value represents the scanned ultraviolet absorption spectrum of the reaction solution after complex formation. The results indicate that the UV absorption spectrum of the prepolymer exhibits a hypochromic effect, which is attributed to the formation of a stable complex between the monomer and the template molecule, thereby affects its absorption characteristics [31].

Furthermore, the successful synthesis of MIP was verified using scanning electron microscopy (SEM), energy-dispersive spectroscopy (EDS), Fourier-transform infrared spectroscopy (FTIR), and specific surface area analysis. Through the scanning electron microscope comparison in Figure 1, it is evident that an imprinting layer was successfully formed on the spherical silica surface. Additionally, the presence of the Zn element confirms the incorporation of quantum dots within the imprinting layer.

**Figure 2:**
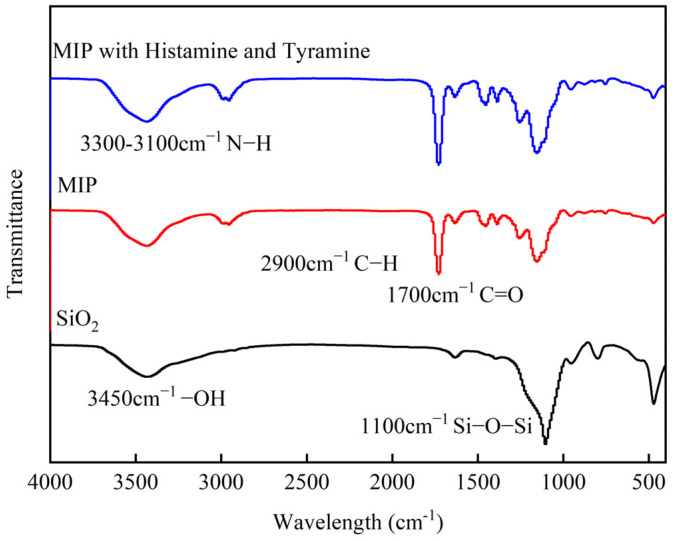
FTIR spectra of SiO_2_, MIP, and MIP with histamine and tyramine.

Figure 2 illustrates the infrared spectra of SiO_2_, MIP, and MIP with bioamine. In all spectra, characteristic absorption peaks at wavenumbers 1100 cm^−1^ (Si-O-Si) and 3450 cm^−1^ (-OH) confirm the existence of SiO_2_ microspheres [32]. Compared to SiO_2_, the peaks at 2900 cm^−1^ and 1700 cm^−1^ correspond to C-H and C=O stretching vibrations in EGDMA [33], respectively, indicating the successful synthesis of imprinted polymers. After bioamine adsorption by MIPs, the transmittance of broad absorption peaks in the range of 3300–3100 cm^−1^ decreased, which can be attributed to the N-H [34] stretching vibrations of bioamines, further validating the successful preparation and effective adsorption capacity of the imprinted polymers.

**Figure 3:**
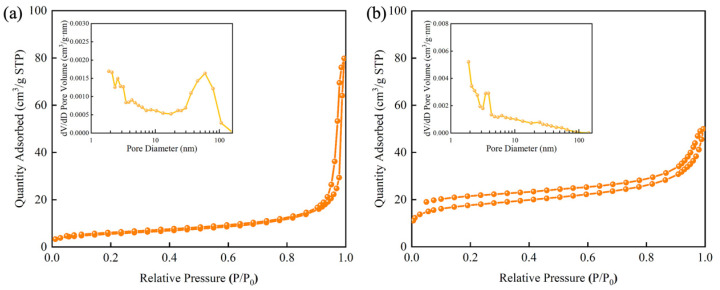
Adsorption–desorption isotherms and pore size distribution of (**a**) SiO_2_ and (**b**) MIP.

According to the nitrogen adsorption–desorption isotherms of SiO_2_ and MIPs (Figure 3), both materials exhibit Type IV [35] isotherm behavior. In the low-pressure region (P/P_0_ < 0.1), the adsorption capacity of MIP is significantly higher than that of SiO_2_, indicating a higher micropore volume and a larger specific surface area [36] in MIP. This enhanced microporosity directly contributes to the increase in the total surface area of MIP. From the analysis of the nitrogen adsorption–desorption isotherms, the BET surface area of SiO_2_ is 19.88 m^2^/g, and the BET surface area of MIP is 65.07 m^2^/g.

### 3.2. Optimization of Polymerization and Detection Conditions

**Figure 4:**
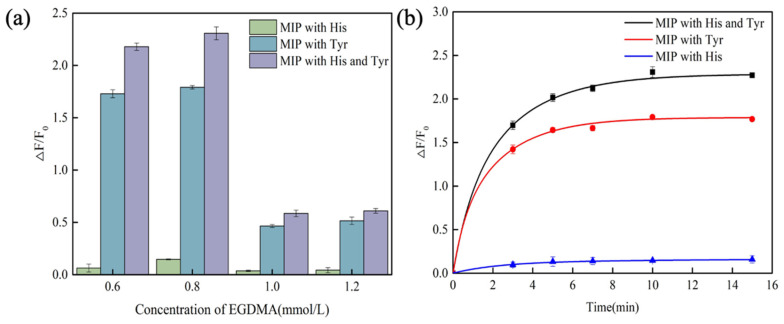
The impact of EGDMA concentration on the fluorescence response of CdSe/ZnS-MIP (**a**). The effect of detection time on CdSe/ZnS-MIP (**b**). (His and Tyr: abbreviations for histamine and tyramine).

In the molecular imprinting polymerization process, the crosslinker stabilizes the interaction between the template molecule and functional monomers [37], forming a highly crosslinked rigid polymer with specific recognition sites for the template molecule [38]. This ensures that the specific recognition sites are retained after the removal of the template molecule. The concentration of the crosslinker plays a critical role in determining the performance of the polymer: an insufficient concentration reduces the crosslinking density [39], compromising the stability of the polymer; conversely, an excessively high concentration leads to over-crosslinking, which causes difficulties in eluting the template molecule and reduces the number of effective imprinting cavities, ultimately decreasing the adsorption performance. Therefore, optimizing the concentration of the crosslinker is a key parameter for preparing high-performance MIPs. At a reaction temperature of 25 °C, MIPs were prepared using different concentrations of EDGMA with 1 mmol/L histamine, tyramine, and their mixed solution. After a 10 min reaction, the fluorescence intensity was measured, as shown in Figure 4a. As the concentration of the crosslinker increases, the fluorescence response of the fluorescent imprinted polymer to histamine, tyramine, and their mixed solution initially increases and then decreases. When the crosslinker concentration reaches 0.8 mmol/L, the fluorescence enhancement rate of the sensor achieves its maximum value, indicating this concentration as the optimal level for the crosslinker.

The detection time is also a critical parameter for evaluating the performance of molecularly imprinted fluorescence sensors. To determine the optimal detection time, we systematically investigated the fluorescence response of the sensor at various binding durations. Fluorescently imprinted polymers reacted with histamine, tyramine, and their mixed solutions for 3, 5, 7, 10, and 15 min, and the corresponding fluorescence enhancement rates were quantitatively measured using a fluorescence spectrometer. As illustrated in Figure 4b, the fluorescence response progressively increased as the reaction time between the sensor and biogenic amines was extended. After 10 min, the fluorescence enhancement rate reached its peak and began to plateau, indicating that the binding at the imprinted sites had approached saturation. Beyond this point, as the reaction time continued to increase, the fluorescence intensity exhibited a slight decline. This phenomenon may be attributed to an increase in non-specific adsorption when the reaction time is excessively prolonged. Consequently, based on these findings, 10 min was identified as the optimal detection duration to ensure the stability and reliability of the fluorescence enhancement rate.

The pH of the solution is also a critical factor influencing the detection capability of molecularly imprinted polymers (MIPs). As depicted in Figure S2, the pH value of the adsorbed solution significantly affects the signal change in the fluorescence response. When the pH is less than 6.0, the fluorescence enhancement efficiency is low due to the weak hydrogen bonding between protonated amino groups and carboxyl groups, or the difficulty in forming hydrogen bonds. When the pH is within the range of 6–8, the fluorescence response of MIPs remains relatively stable, and a pH of 7.0 is selected as the optimal detection condition.

### 3.3. Sensitivity of CdSe/ZnS-MIP for Histamine and Tyramine

To evaluate the fluorescence response of the prepared sensor toward two biogenic amines, fluorescence spectra were measured for solutions containing histamine and tyramine mixed in equal proportions at identical concentrations, as well as for individual solutions of histamine and tyramine. As the concentration of biogenic amines increased, the fluorescence intensity of CdSe/ZnS-MIP at 620 nm was progressively enhanced. Furthermore, the fluorescence response signals were fitted, and the results indicated that tyramine had a more pronounced influence on the adsorption and detection process of MIP compared to histamine. The fluorescence response of CdSe/ZnS-MIP to the mixed solution of biogenic amines was subjected to linear fitting (Figure S3). The results showed that the limit of detection (LOD), calculated based on three times the standard deviation of the blank control (3σ), was 14.57 μmol/L. This value exhibited satisfactory detection performance when compared to the minimum detectable concentration of biogenic amines in fermented foods as specified by the EU.

**Figure 5:**
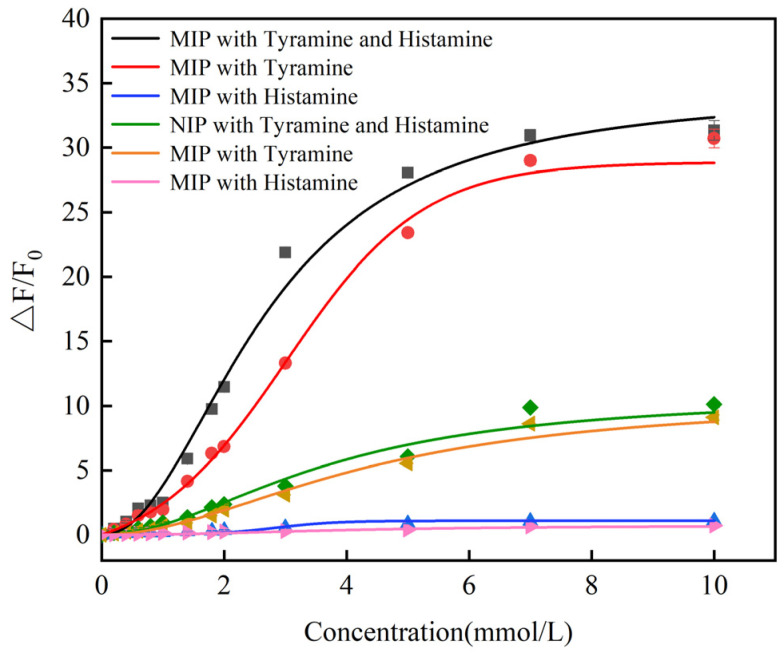
The detection of histamine and tyramine and mixed solutions at the same concentration by CdSe/ZnS-MIP and NIP.

Figure 5 demonstrates that the signal of histamine is significantly lower than that of tyramine. In the prior experiments, we synthesized a double-template imprinted polymer without CdSe@ZnS and quantified its adsorption capacity using high-performance liquid chromatography (HPLC). Within the concentration range of 0–0.3 mmol/L for histamine and tyramine, the adsorption behavior of these two biogenic amines by the molecularly imprinted polymer (MIP) is illustrated in Figure S4, with tyramine exhibiting a higher adsorption capacity compared to histamine. Additionally, the fluorescence responses of the two biogenic amines were evaluated using CdSe@ZnS quantum dots. The fluorescence response of the quantum dots toward tyramine was stronger than histamine, which explains why the fluorescence response of tyramine surpasses that of histamine in CdSe/ZnS-MIP.

### 3.4. Selectivity and Stability of CdSe/ZnS-MIP for Histamine and Tyramine

**Figure 6:**
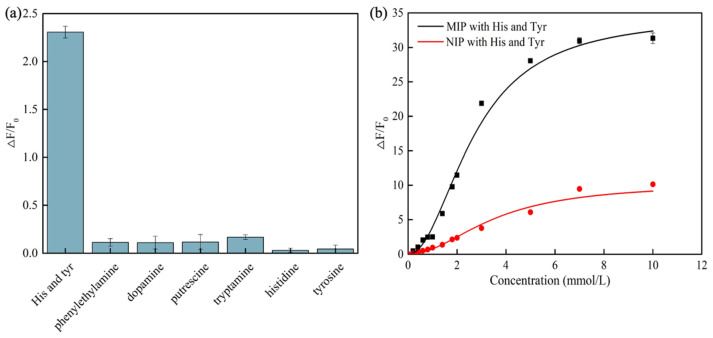
Selectivity of CdSe/ZnS MIP and CdSe/ZnS NIP towards histamine, tyramine, and structural analogs (**a**). Application of CdSe/ZnS-NIP in the detection of histamine and tyramine (**b**). (His and Tyr: abbreviations for histamine and tyramine).

The aim of the selective recognition experiment was to evaluate whether the molecularly imprinted polymer (MIP) could effectively distinguish and specifically adsorb target molecules while exhibiting minimal interaction with other biogenic amines and structural analogs. Under identical detection conditions, the adsorption performance of CdSe/ZnS-MIP was assessed for the mixed solution of histamine and tyramine as well as their structural analogs, including tryptamine, dopamine, phenylethylamine, putrescine, histidine, and tyrosine. As illustrated in Figure 6a, at the same concentration, the fluorescence response of CdSe/ZnS-MIP to the histamine–tyramine mixture was significantly stronger compared to its responses to tryptamine, dopamine, phenylethylamine, putrescine, histidine, and tyrosine. This result demonstrates the high selectivity of the sensor. This phenomenon can be attributed to the absence of specific imprinted cavities or recognition sites on the surface of CdSe/ZnS-MIP that could bind to these structural analogs, leading to negligible fluorescence enhancement. This marked difference further validates the superior selectivity of CdSe/ZnS-MIP in detecting histamine and tyramine.

The selective fluorescence response of MIP to the analyte can be attributed to the specific adsorption and recognition of the analyte by the imprinted cavities formed during the preparation process. NIP refers to the process of preparing imprinted polymers without adding any template molecules. Therefore, the surface of NIPs does not contain cavities that can combine with template molecules. While NIPs lack the selective adsorption ability of MIPs, they still exhibit physical adsorption characteristics. As shown in Figure 6b, the fluorescence enhancement of NIPs is significantly lower than that of MIPs. Specifically, the fluorescence enhancement rate of MIPs is 3.1 times higher than that of NIPs, which confirms the successful preparation and outstanding specificity of the sensor.

As previously discussed in Section 3.3, MIP exhibits a higher adsorption capacity for tyramine compared to histamine, while CdSe@ZnS quantum dots demonstrate a stronger fluorescence response to histamine relative to tyramine. Consequently, regarding histamine detection, although the response value of NIP to the bioamine mixture solution is higher than that of MIP to histamine alone, the overall response of NIP to histamine remains lower than that of MIP.

Finally, the repeated adsorption capacity of CdSe/ZnS-MIP was evaluated, as shown in Figure S5a, five adsorption–desorption cycles were performed with 1 mmol/L biogenic amines as the target, and CdSe/ZnS-MIP still had good detection ability for biogenic amines. Furthermore, by assessing multiple batches of MIPs prepared at different times, it was observed that the detection performance of MIPs remained essentially stable over 30 days.

Compared with single-template fluorescent-imprinted polymers [40], double-template fluorescent-imprinted polymers exhibit a broader linear range for the detection of histamine and tyramine, extending from 20–80 μM for histamine to 0.01–5 mmol/L for histamine and tyramine. This may be attributed to the interaction between high-affinity binding sites and low-affinity binding sites for the two template molecules within the imprinting cavity, and the different detection performance of the two template molecules may be optimized by adjusting the ratio of template molecules or selecting different types of quantum dots. The broader linear range represents a significant advantage in the rapid detection of biogenic amines in complex food matrices.

### 3.5. Application of CdSe/ZnS-MIP in Cheese Samples

To evaluate the accuracy of CdSe/ZnS-MIP in detecting two biogenic amines in real food samples, cheese samples spiked with known concentrations were analyzed. The recovery results are presented in Table 1, with recovery rates ranging from 99.46% to 103.02% and relative standard deviations (RSDs) ranging from 3.73% to 6.16%. Furthermore, the method was validated by comparison with HPLC, confirming its reliability. Therefore, the CdSe/ZnS-MIP-based detection method can be considered a valuable tool for detecting biogenic amines and ensuring the safety of dairy products. Given the complexity of cheese sample pretreatment, these results also demonstrate the potential of the sensor for the rapid and stable detection of biogenic amines in complex food matrices.

### 3.6. Comparison of CdSe/ZnS-MIP with Other Methods

Compared with the research on the detection of biogenic amines by molecular imprinting technology (Table 2), the developed CdSe/ZnS-MIP sensor provides a simplified procedure for the simultaneous detection of two biogenic amines, Compared with imprinted polymers that respond to the specific fluorescence of bioamines through the incorporation of ionic liquids or o-phthaldialdehyde, the proposed sensor reduces the detection time to 10 min and decreases the detection limit to 14.57 μmol/L. This analytical approach is cost-effective and eliminates the need for complex sample pretreatment steps such as derivatization or the use of specialized instruments. Moreover, compared with existing molecularly imprinted polymer-based detection methods, the proposed CdSe/ZnS-MIP exhibits a significantly faster response time.

## 4. Conclusions

In this study, we developed a dual-template fluorescence imprinted sensor based on MIP, integrated with CdSe/ZnS quantum dots, which enables the rapid and specific recognition of histamine and tyramine in complex food matrices. By optimizing the concentration of functional monomers and reaction time during the polymerization process, the detection performance of the CdSe/ZnS-MIP sensor was significantly enhanced. The results demonstrated that this sensor can achieve the rapid and quantitative detection of the two most abundant and toxic biogenic amines within 10 min, over the range of 0.01–10 mmol/L, with a limit of detection (LOD) of 14.57 μmol/L. Furthermore, the preparation of the sensor is cost-effective and does not require expensive instruments, addressing the limitations of traditional methods such as high costs, complex operational procedures, long processing times, and environmental pollution caused by the extensive use of organic solvents. Finally, the developed imprinted polymer fluorescence sensor is capable of analyzing complex real samples, such as cheese with high fat and protein content. This multi-template sensing technology offers a more time-efficient, environmentally friendly, and cost-effective approach for biogenic amine detection, paving the way for promising future applications.

## Figures and Tables

**Table 1:** Determination of histamine and tyramine in cheese.

Sample	Spiked/(mg/kg)	Found/(mg/kg)	RSD (%)	HPLC/ (mg/kg)	RSD(%)	Recovery (%)
1	0	-	-	15.31 ± 0.22	1.42	-
2	318	320.97 ± 11.98	3.73	311.57 ± 2.88	0.92	103.02 ± 3.85
3	636	651.53 ± 36.38	5.58	655.06 ± 29.82	4.55	99.46 ± 5.55
4	954	1006.3 ± 62.02	6.16	992.05 ± 43.25	4.4	101.44 ± 6.25

**Table 2:** Comparison of different detection methods.

Biogenic Amines	Method	Sensor	Detection Time (min)	LOD	Detected Sample	Ref.
Histamine	Fluorescence spectroscopy	o-phthaldialdehyde-MIP	240	1.8 μmoL/L	Milk	[23]
Histamine	Fluorescence spectroscopy	MXene-MIP	20	21.9 nmoL/L	Fish	[26]
Tyramine	Electrochemical measurements	MIP	-	159 μmoL/L	Cheese	[41]
Histamine	ELISA	mimic enzyme labeled with histamine antibody	190	0.50 mg/kg	Fish	[42]
Histamine	Quartz crystal microbalance	MIP	20	5 nmoL/L	Tunny	[43]
Histamine	Fluorescence spectroscopy	QDs-Ionic liquids -MIP	390	110 μmoL/L	Fish	[29]
Histamine and Tyramine	Fluorescence spectroscopy	CdSe/ZnS-MIP	10	14.57 μmoL/L	Cheese	This method

## Data Availability

The original contributions presented in this study are included in the article/Supplementary Materials. Further inquiries can be directed to the corresponding author.

## Supplementary Materials

The following supporting information can be downloaded at: https://www.mdpi.com/article/10.3390/foods14091475/s1, Figure S1: The UV absorption spectra of functional monomers and template molecules; Figure S2: The effect of pH on the fluorescence response of CdSe/ZnS-MIP; Figure S3: Fluorescence response of CdSe/ZnS-MIP to different concentrations of histamine and tyramine; Figure S4: The adsorption and fluorescence responses of CdSe/ZnS and MIP to biogenic amines; Figure S5: The Repeatability and Stability of CdSe/ZnS-MIP for Biogenic Amine Detection; Chromatographic condition. Table S1: Mobile Phase Elution Gradient Conditions.
