# Supplementary material for: A Molecularly Imprinted Fluorescence Sensor for the Simultaneous and Rapid Detection of Histamine and Tyramine in Cheese
Source: Foods. 2025 Apr 23;14(9):1475. doi: 10.3390/foods14091475 (PMC12072070; doi:10.3390/foods14091475)
Supplement: Supplementary file 1 [file foods-14-01475-s001.zip › foods-3568308-supplementary.pdf]

Supplementary Information

A Molecularly Imprinted Fluorescence Sensor for Simultaneous and Rapid Detection of Histamine and Tyramine in Cheese

2.1 Chromatographic condition

A Hypeisil ODS column (150 mm × 4.6 mm, 5 μm) was used for separation, and the ultraviolet detection wavelength was set at 254 nm. The Column temperature was kept at 35℃, The injection volume was 20 μL, and the flow rate was kept at 0.8 mL/min. The gradient elution procedure is shown in Table S1.

Table S1 Mobile Phase Elution Gradient Conditions

Time(min)	flow rate(mL/min)	Mobile phase A(%)	Mobile phase B(%)
0	0.8	60	40
15	0.8	85	15
18	0.8	100	0
23	0.8	60	40
25	0.8	60	40

Figure:

Figure S1 The UV absorption spectra of functional monomers and template molecules

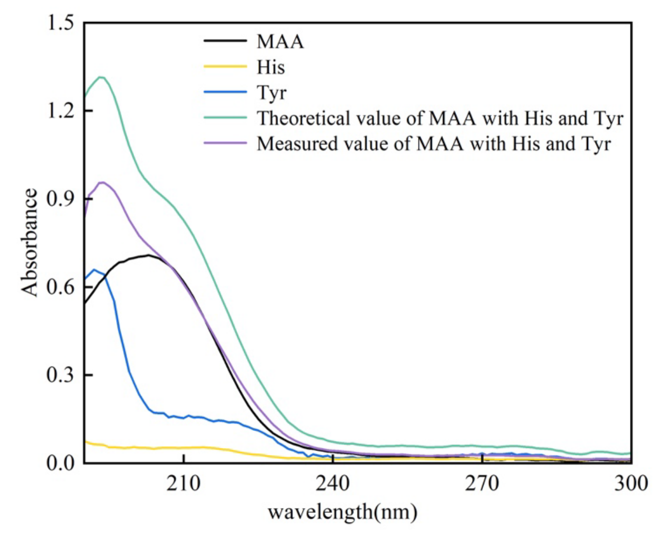

Figure S1 UV absorption spectra of functional monomers and template molecules

Figure S2 The effect of pH on the fluorescence response of CdSe/ZnS-MIP

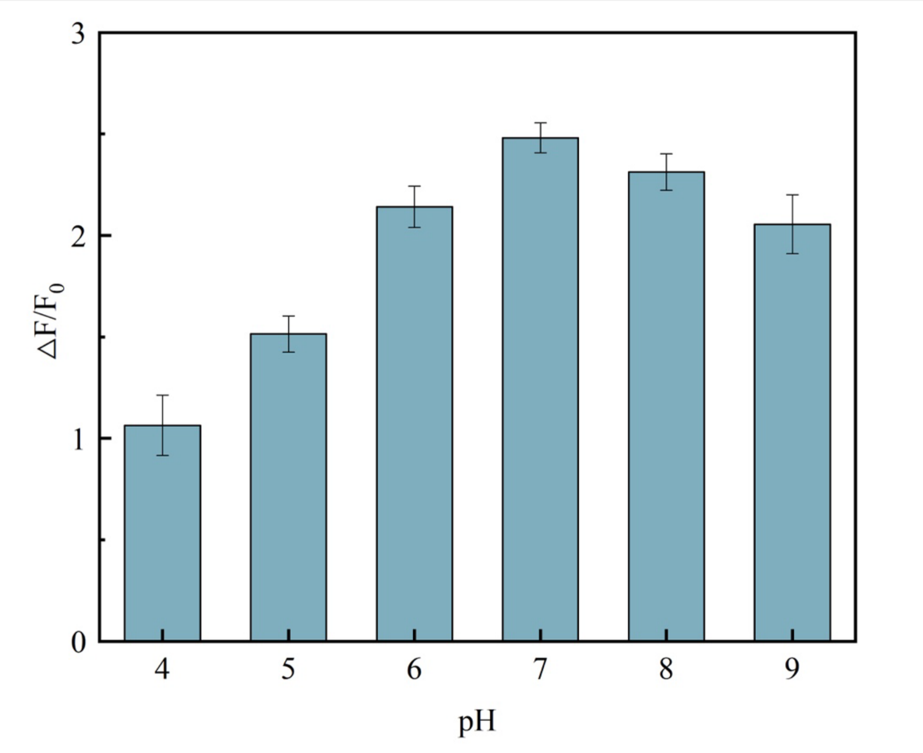

Figure S2 Fluorescence response of CdSe/ZnS-MIP at different pH

Figure S3 Fluorescence response of CdSe/ZnS-MIP to different concentrations of histamine and tyramine

The detection values of CdSe/ZnS-MIP under different bioamine concentration gradients were linearly fitted. The linear of the sensor is ranges from 0.01- 5mM.

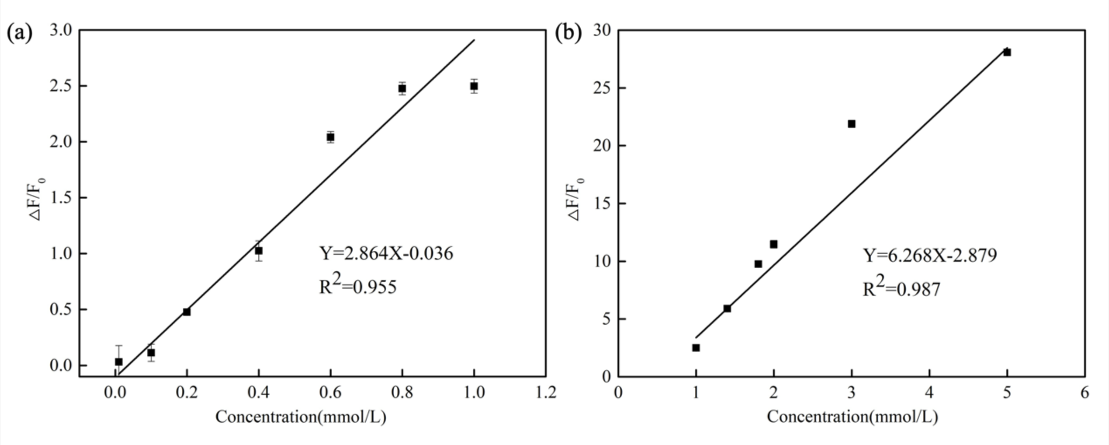

Figure S3 CdSe/ZnS-MIP linear standard curve for different concentrations of mixed solution containing histamine and tyramine:(a) The concentration range from 0.01 to 1 mM; (b) The concentration range from 1 to 5 mM;

Figure S4 The adsorption and fluorescence responses of CdSe/ZnS and MIP to biogenic amines

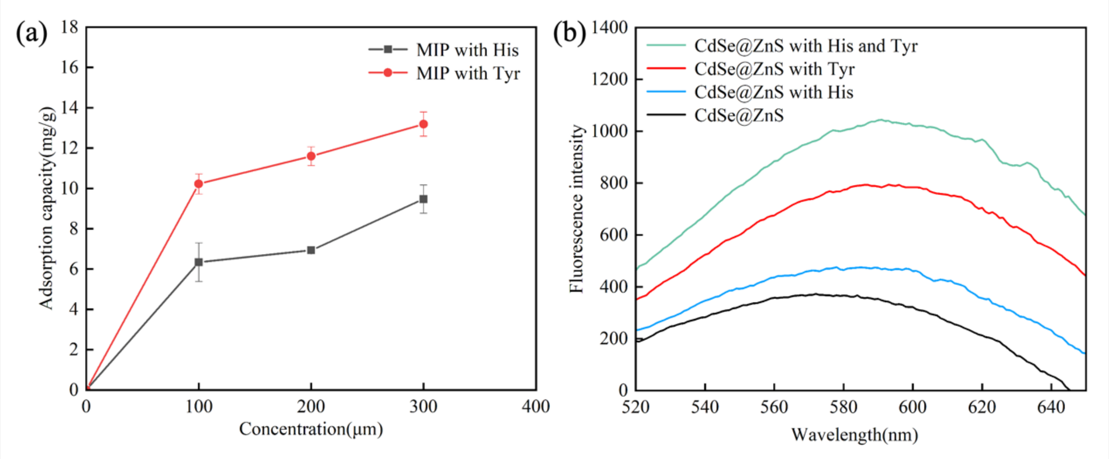

Figure S4. (a)Adsorption capacity of MIP for histamine and tyramine respectively; (b) Fluorescence response of CdSe@ZnS to histamine and tyramine

Figure S5 The Repeatability and Stability of CdSe/ZnS-MIP for Biogenic Amine Detection

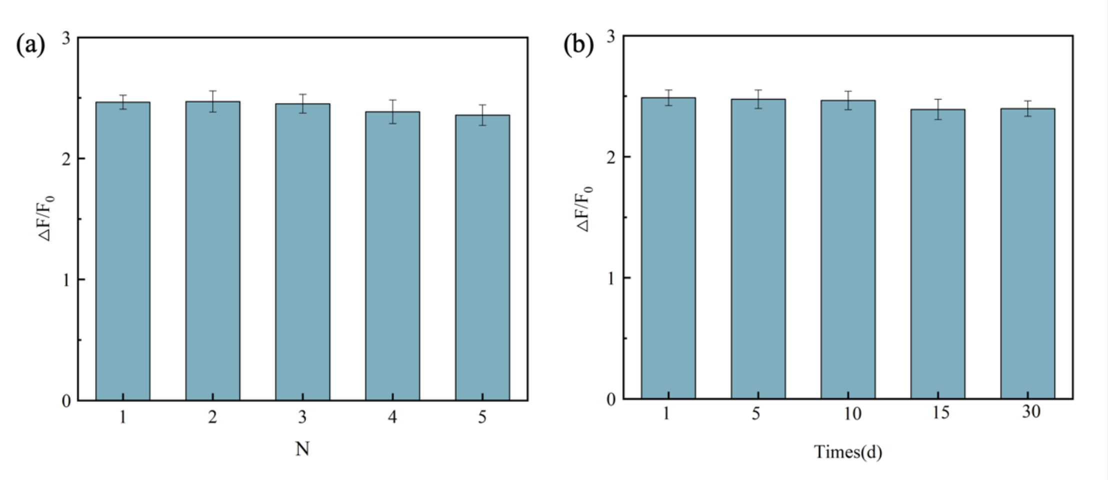

Figure S5 (a)The repeated adsorption capacity of CdSe/ZnS-MIP; The Stability of CdSe/ZnS-MIP.
